# An Optimal Shaped Sensor Array Derivation

**DOI:** 10.3390/mi14061154

**Published:** 2023-05-30

**Authors:** Marco Dibiase, Luca De Marchi

**Affiliations:** 1 Department of Computer Science and Engineering, University of Bologna, 40136 Bologna, Italy; 2 Department of Electrical, Electronic, and Information Engineering “Guglielmo Marconi”, University of Bologna, 40136 Bologna, Italy

**Keywords:** optimal shaped sensors, Direction of Arrival, DoA, Guided Waves, Lamb Waves, piezoelectric sensors, sensor array, sensors cluster, Shaped Sensors Optimal Cluster, Calculus of Variations

## Abstract

In Structural Health Monitoring (SHM) applications, the Direction of Arrival (DoA) estimation of Guided Waves (GW) on sensor arrays is often used as a fundamental means to locate Acoustic Sources (AS) generated by damages growth or undesired impacts in thin-wall structures (e.g., plates or shells). In this paper, we consider the problem of designing the arrangement and shape of piezo-sensors in planar clusters in order to optimize the DoA estimation performance in noise-affected measurements. We assume that: (i) the wave propagation velocity is unknown, (ii) the DoA is estimated via the time delays of wavefronts between sensors, and (iii) the maximum value of the time delays is limited. The optimality criterion is derived basing on the Theory of Measurements. The sensor array design is so that the DoA variance is minimized in an average sense by exploiting the Calculus of Variations. In this way, considering a three-sensor cluster and a monitored angles sector of 90°, the optimal time delays–DoA relations are derived. A suitable re-shaping procedure is used to impose such relations and, at the same time, to induce the same spatial filtering effect between sensors so that the sensor acquired signals are equal except for a time-shift. In order to achieve the last aim, the sensors shape is realized by exploiting a technique called *Error Diffusion,* which is able to emulate piezo-load functions with continuously modulated values. In this way, the *Shaped Sensors Optimal Cluster* (SS-OC) is derived. A numerical assessment via Green’s functions simulations shows improved performance in DoA estimation by means of the SS-OC when compared to clusters realized with conventional piezo-disk transducers.

## 1. Introduction

In the Structural Health Monitoring (SHM) field [1,2,3,4], passive piezoelectric sensors, permanently bonded on the structure, are able to detect and localize an Acoustic Source (AS) due to damages growth or undesired impacts with foreing objects. Therefore, they allow monitoring in real time. An increasing demand of such passive SHM systems comes from several application contexts, e.g., industrial, civil, automotive, etc., thanks to their high potential to improve the safety, predict incipient failures, estimate the remaining useful life and reduce the maintenance costs. Novel monitoring approaches are particularly requested by the aeronautical and aerospace fields for weight saving of the monitoring systems together to the associated cabling. As a consequence, wireless and battery powered-embedded devices for data acquisition, pre-processing and transfer to a Central Unit (CU) are to be used. In turn, a low-power consumption for the in-site data processing and data transferring is needed. A further complication is given by the ultrasonic propagation within thin-wall structures, such as shells of an aircraft wing or fuselage. In these thin-wall plates, AS generate Guided stress Waves (GW), such as Lamb Waves [5] or Shear Horizontal (SH) waves [1]. They are characterized to be dispersive, i.e., with a non-linear relation between the wave-number and the frequency, and multi-modal, i.e., different wave modes can propagate, each one with a different wave number–frequency relation (*dispersion curve*). Although different AS location methods were proposed and validated at the laboratory scale, such as inverse methods [6,7,8] and hyperbolic positioning [9,10], they rarely satisfy the previous constraints imposed for the field deployment. Indeed, the inverse methods require a very accurate modeling of ultrasonic propagation and a high computational cost to simulate the inverse propagation. Therefore, they are not suitable for embedded applications or to transfer a small amount of data to the CU. On the other hand, the hyperbolic positioning requires a large number of sensors in order to reduce the AS estimation uncertainty. Furthermore, an accurate synchronization barely is achievable via wireless systems.

As result, multiple clusters of closely located sensors, able to estimate the AS location via the wavefront Direction of Arrival (DoA) estimation on each one, are often the only feasible solution. Indeed, these methods have a computational cost compatible with embedded systems and do not require synchronization between distant sensors.

Different strategies which use one or more clusters for the AS estimation have been studied and tested [11,12,13,14]. Among these, the first one, known as Multiple Signal Classification (MUSIC) [11], allows estimating up to N-1 DoAs due to different sources with N-sensor arrays. Modified MUSIC algorithms allow detecting and estimating the DoAs of Lamb Waves [15]. However, MUSIC algorithms are heavily limited by the assumption of accurate knowledge or estimation of wave velocity. Therefore, an additional iterative wave velocity estimation procedure is needed (as shown in [16]), which increases consistently the computational cost. Therefore, for the unknown velocity assumption considered in this work, the MUSIC algorithms are unsuitable.

In the works [12,13], a single cluster is used to locate not only the AS angle but also its distance. Nevertheless, in order to estimate the AS distance, they require detecting two modes (typically the A0 and S0 Lamb modes) and computing their time difference on the sensors by knowing their different wave velocities. Therefore, they are inadequate for monitoring systems which are to be applied on any material with unknown properties. Conversely, the technique proposed by Kundu [14] is suitable even when one only wave mode is detected. This is the case of an AS generated by an impact, when, typically, the Lamb Waves A0 fundamental mode has a much higher amplitude with respect to other guided modes. Such a technique uses—at least—two closely spaced sensors clusters placed apart to estimate multiple DoAs and to locate sources both in isotropic and slightly anisotropic plates.

The DoA estimation performance of a simple cluster of three circular sensors placed on the vertices of an isosceles right triangle, with unknown wave velocity, was investigated in [17,18]. The DoA estimation is performed by means of the Differences in Time of Arrival (DToAs) estimations via simple Cross-Correlation (CC) procedures. In this work, this cluster will be referred to as *Standard Cluster* (SC). In [19,20,21], it was shown that it is possible to use multiple clusters to estimate the AS localization even in the case of heavily anisotropic structures (i.e., characterized by elliptical or rhomboidal wavefronts).

Although using the SC for AS location was well-validated at laboratory scale, it is well-known that in realistic field deployment, undesired signal components, i.e., noise, can be generated by different physical phenomena [22]: structural vibration (e.g., due to turbulence on an aircraft), scattered wave-field, noisy acquisition channels, or noisy electronic devices.

Research efforts have been already aimed to address the noise issue in DoA estimation. In [23], Oktel and Moses proposed a disk-sensors cluster design procedure to increase the DoA estimation performance in presence of noise. It was based on the Bayesian approach (or *global*) of the Cramér–Rao bound (CRB), which depends on the sensor positioning and defines the lower bound of any unbiased estimator. However, in that work, the wave velocity is supposed to be known. Therefore, in a previous work [24], a modification of the approach presented in [23] was proposed, which avoids the previous assumption, in order to find the disk sensor positioning for the optimal DoA estimation, when, at the same time, the accuracy loss due to the unknown wave velocity is minimized.

In other previous works [25,26], the design paradigm of the sensor clusters was radically changed by considering sensors with different shapes with the ultimate aim to increase the DoA estimation performance. More specifically, in [25], a novel design strategy was proposed to control the wave-number filtering effect induced by sensors differently shaped, considering a cluster of just two sensors for guided wave mode detection. The proposed procedure allows using again CC procedures for time delays estimation, ensuring same frequency responses except for phase shifts, i.e., delays in time.

In [26], a three-shaped-sensor cluster was proposed to estimate the DoA without knowing the wave velocity. Such a strategy is capable of reducing the estimation uncertainty, but the shaped sensors optimization was not addressed. Conversely, in the present work, an optimality criterion and a further improvement in shaped sensor synthesis are proposed and numerically validated.

In greater detail, the innovations proposed in this work are illustrated as follows. In Section 2, the optimality criterion based on the Theory of Measurements is exploited to find the optimal shapes of three-sensors cluster. It is assumed that the wave velocity is unknown, the DoA is estimated via the time delays of wavefronts between sensors, and the maximum value of the DToAs is limited. The sensor array design is so that the DoA variance is minimized in an average sense by exploiting the Calculus of Variations for functionals [27]. In this way, considering a monitored angles sector of 90° as a representative case of different realistic scenarios, the optimal time delays–DoA relations are derived together to the variance improvement with respect to the disk sensors SC one. In Section 3, the re-shaping procedure based on the Radon Transform (RT) tool, already employed in [25,26] to address the different wave-number filtering among differently shaped sensors, is in more detail reviewed, providing the physical meaning of the sensors RT, i.e., an equivalent 1D piezo-load distribution able to provide the same effect of the in plane 2D piezo-load. A novel quantization technique is illustrated and applied to generate a dithered shaped piezo-sensor able to emulate *shape functions* continuously modulated in values. It is called *Error Diffusion* [28] and it is derived by the image processing techniques. Via the previous techniques, three piezo-loads are derived by defining the, so-called, *Shaped Sensors Optimal Cluster* (SS-OC). In Section 4, different DoA estimators based on four different DToAs estimator are illustrated and discussed. They are employed in Section 5 on synthetic noisy signals in order to assess the expected 50% reduction of the Standard Deviation (SD) in DoA estimations via the SS-OC with respect to the SC of piezo-disks when the acquired signals are affected by noise. The numeric results are examined according to the employed estimator, the sensor quantization non-idealities, the signal bandwidth and the wave velocity. In Section 6, the numeric results are discussed in terms of comparison between the SC and the *Disk Sensors Optimal Cluster* (DS-OC), which was proposed in [24]. The conclusions are given in Section 7.

## 2. Optimal Time Delays–DoA Functions Derivation

Let us assume that a sensor array consists of three arbitrarily shaped sensors: P1, P2, and P3. Moreover, let us suppose that a single co-planar far-field source generates the wave-field impinging the three-sensors array from DoA θ. The output signal of the *i*th sensor can be expressed as:

(1) $$x_{i}\left(t\right)=s\left(t-d_{i1}\left(\theta \right)\right)+n_{i}\left(t\right)$$

where s(t) is the wave signal at a reference point near the array, assumed, without loss of generality, to be coincident with the location of the first element in the array, P1. $n_{i}\left(t\right)$ is the additive sensor noise at the *i*th sensor. $d_{i1}\left(\theta \right)$ is the time delay at the *i*th sensor with respect to the reference point P1. Therefore, $d_{11}\left(\theta \right)$ is equal to 0.

In order to estimate the DoA, one can first estimate the vector of DToAs $d={\left[d_{21},d_{31}\right]}^{T}$. The following assumptions are also considered:

1. The sensors are closely spaced so that the wave amplitude gradient across the array is negligible.
2. The bandwidth of the acquired signals is narrow and the sensors spacing is small so that the effect of the wave dispersion is negligible. In case of wide-band signals, the previous assumption can still be satisfied by decomposing the signals with narrowband filter banks.
3. The wave number filtering effect (induced by the sensor shaping) will be imposed to be equal on the different sensors by using an appropriate re-shaping procedure which will be illustrated in the following section. In this way, the well-known (Generalized) Cross-Correlation (GCC) procedures can be used to estimate the DToAs.

Observe that in case of disk-shaped sensors, the time delay–DoA relations, $d_{i1}\left(\theta \right)$, are prescribed only by the sensor positioning. Conversely, the proposed strategy allows designing arbitrary $d_{i1}\left(\theta \right)$ relationships by varying the sensors shape.

In this sense, let us consider two arbitrary relationships between the DToAs ($d_{21}$ and $d_{31}$) and the wave direction of propagation θ in the following form:

(2) $$d_{21}\left(\theta \right)=\frac{\rho_{21}\left(\theta \right)}{v}\;\;,\;\;d_{31}\left(\theta \right)=\frac{\rho_{31}\left(\theta \right)}{v}$$

where $\rho_{21}\left(\theta \right)$ and $\rho_{31}\left(\theta \right)$ are arbitrary functions and *v* is the wave velocity. The last one is regarded as a constant parameter under the assumption of low dispersion, as better specified in Section 3. Observe that the physical dimensions of the two functions $\rho_{21}\left(\theta \right)$ and $\rho_{31}\left(\theta \right)$ correspond to spatial shifts. Indeed, they represent the Differences in Distance of Arrival (DDoAs), which are defined by sensor arrangement and shape. The aim is thus to find the optimal estimator in the form:

(3) $$\hat{\theta }=f\left(\frac{{\hat{d}}_{21}}{{\hat{d}}_{31}}\right)$$

which is necessary to estimate the DoA θ without knowing the wave velocity *v*. The quantities ${\hat{d}}_{21},{\hat{d}}_{31}$ indicate the estimators (i.e., random variables (r.v.)) used for the DToAs.

The optimal estimator, for estimation functions such as (3), is the one that minimizes its variance, $\sigma_{\hat{\theta }}^{2}$, provided by the Theory of Uncertainty Propagation for measurements [29]. According to this theory, if we assume an unbiased estimation function *f* (so that the Mean Square Error, $e_{ms}$, is equal to $\sigma_{\hat{\theta }}^{2}$) then, by using the first-order Taylor’s expansion of estimation function *f* and neglecting the correlation between ${\hat{d}}_{21},{\hat{d}}_{31}$, the variance $\sigma_{\hat{\theta }}^{2}$ can be written as:

(4) $$\begin{array}{c} \sigma_{\hat{\theta }}^{2}=e_{ms}=E\left[{\hat{\theta }}^{2}\right]-\theta^{2}=\\ \;\;\;\;\;\;\;={\left.{\left(\frac{\partial f}{\partial {\hat{d}}_{21}}\right)}^{2}\right|}_{E\left[{\hat{d}}_{21}\right],E\left[{\hat{d}}_{31}\right]}\sigma_{{\hat{d}}_{21}}^{2}+{\left.{\left(\frac{\partial f}{\partial {\hat{d}}_{31}}\right)}^{2}\right|}_{E\left[{\hat{d}}_{21}\right],E\left[{\hat{d}}_{31}\right]}\sigma_{{\hat{d}}_{31}}^{2} \end{array}$$

where the terms $\sigma_{{\hat{d}}_{21}}^{2},\sigma_{{\hat{d}}_{31}}^{2}$ are the DToA variances. Such quantities are related to the DToAs estimator performance and, considering a small perturbation analysis, to the signal and noise spectra. If an efficient DToAs estimator is used, they equal the diagonal terms of the time delays CR Matrix-Bound (CRMB) [30].

The worst-case variance value is given by:

(5) $$\sigma_{\hat{\theta }-WorstCase}^{2}=\left({\left(\frac{\partial f}{\partial {\hat{d}}_{21}}\right)}^{2}+{\left(\frac{\partial f}{\partial {\hat{d}}_{31}}\right)}^{2}\right)\sigma_{d-Max}^{2}$$

where $\sigma_{d-Max}^{2}=\max\left[\sigma_{{\hat{d}}_{21}}^{2},\sigma_{{\hat{d}}_{31}}^{2}\right]$. The aim is then to minimize (5) on average over all the expansion points $\;E\left[{\hat{d}}_{21}\right]=d_{21},E\left[{\hat{d}}_{31}\right]=d_{31}$. Therefore, the aim is to minimize the following integral:

(6) $$J\left[f\right]=\frac{\sigma_{d-Max}^{2}}{\Omega }\int \int_{\Omega }\left(f_{d_{21}}^{2}+f_{d_{31}}^{2}\right)dd_{21}dd_{31}$$

where, to simplify the notation, $f_{d_{21}}=\frac{\partial f}{\partial d_{21}},f_{d_{31}}=\frac{\partial f}{\partial d_{31}}$. The integral is computed over Ω, i.e., the variation range of $E\left[{\hat{d}}_{21}\right],E\left[{\hat{d}}_{31}\right]$. It can be observed that Equation (6) is configured as a functional, J[f] which can be minimized with the tools developed within the Calculus of Variations (CoV) theory, which is widely used in multiple Physics fields [31]: from classical Mechanics, with the Principle of Least Action, to Optics, with the Maupertuis’s principle; from Quantum Mechanics, with the Feynman integrals, to the advanced physics of elementary particles interaction.

The CoV theory allows deriving compact expressions, via the integrand function, namely the Lagrangian function. In our case, the Lagrangian function is simply expressed by $\sigma_{\hat{\theta }-WorstCase}^{2}$.

The CoV provides the means to find *extremal functions*, which are the candidate functions to be *extremant*, i.e., to minimize or maximize the generic functional J[f], by exploiting the Euler–Lagrange differential equation for functionals [27].

It is worth noting that the functional (6) depends on a function *f* of two variables. Observe that the CoV requires that a functional *J* depends on functions of independent variables. Therefore, we will start by considering $d_{21}$, $d_{31}$ as two independent variables, as if they can describe a surface on the $d_{21}-d_{31}$ 2D-plane. Then, the function *f* within the Euler–Lagrange equation will be written as a function of a single (independent) variable, i.e., the time-delays ratio. The time delays $d_{21}$,$d_{31}$ dependence will be resumed *a posteriori* by selecting two appropriate functions providing the found extremal function *f* (namely, describing a curve on the 2D-plane $d_{21}-d_{31}$ via a two-equation parameterization of the independent variable θ).

It is possible to formally prove that the minimization of the functional (6) (considering mobile boundary conditions) is equivalent to the minimization of a functional in the form $J[d_{21}\left(\theta \right),d_{31}\left(\theta \right)]$, namely depending by two functions of a single (independent) variable θ. To this aim, the Lagrangian function, neglecting the constant terms, can be written as:

(7) $$\begin{array}{c} {\left(\frac{\partial f}{\partial d_{21}}\right)}^{2}+{\left(\frac{\partial f}{\partial d_{31}}\right)}^{2}={\left(\frac{\partial f}{\partial \eta }\frac{1}{d_{31}}\right)}^{2}+{\left(-\frac{\partial f}{\partial \eta }\frac{d_{21}}{d_{31}^{2}}\right)}^{2}\\ ={\left(\frac{\partial f}{\partial \eta }\right)}^{2}\left(\frac{d_{31}^{2}\left(\theta \right)+d_{21}^{2}\left(\theta \right)}{d_{31}^{4}\left(\theta \right)}\right) \end{array}$$

where η=d21d21d31d31 was posed. By using the theorem of the inverse function derivative, the term ∂f∂f∂η∂η can be rewritten as:

(8) $$\frac{\partial f}{\partial \eta }=\frac{1}{\frac{d\left(\frac{d_{21}\left(\theta \right)}{d_{31}\left(\theta \right)}\right)}{d\theta }}=\frac{d_{31}^{2}\left(\theta \right)}{d{}_{21}^{\prime }\left(\theta \right){d^{}}_{31}\left(\theta \right)-d_{21}\left(\theta \right)d{}_{31}^{\prime }\left(\theta \right)}$$

Therefore:

(9) $$J\left[d_{21},d_{31}\right]=\frac{2\sigma_{d-Max}^{2}}{\theta_{0}}\int_{-\theta_{0}}^{\theta_{0}}\frac{d_{31}^{2}\left(\theta \right)+d_{21}^{2}\left(\theta \right)}{{\left(d{}_{21}^{\prime }\left(\theta \right){d^{}}_{31}\left(\theta \right)-d_{21}\left(\theta \right)d{}_{31}^{\prime }\left(\theta \right)\right)}^{2}}d\theta$$

Observe that the last functional proved the equivalence with Equation (6) and, at the same time, allows selecting a prior probability function f(θ) which differs by a uniform density function (implicit in (9)). However, the functional in Equation (9) involves a Euler–Lagrange equation more complicated with respect to the functional (6) one. Therefore, to proceed, we consider the functional in the previous form (6).

Given an integrand function for a functional of form $F(x,y,f\left(x,y\right),f_{x},f_{y})$, Euler’s equation is given by:

(10) $$F_{f}=\frac{\partial F_{f_{x}}}{\partial x^{}}+\frac{\partial F_{f_{y}}}{\partial y}$$

where $F_{f}=\frac{\partial F}{\partial f},F_{f_{x}}=\frac{\partial F}{\partial f_{x}}$ and $F_{f_{y}}=\frac{\partial F}{\partial f_{y}}$. It follows that the following system must be solved:

(11) $$\left\{\begin{array}{c} f_{d_{21}d_{21}}+f_{d_{31}d_{31}}=0\\ +\mathrm{boundary}\;\mathrm{conditions}:f={\left.g(d_{21},d_{31})\right|}_{\partial \Omega } \end{array}\right.$$

The first of (11) is the Laplace equation which is solved with harmonic functions. More specifically, we seek solutions in the form $f(d_{21}/d_{31})$ which limit the possible set of admissible domains for Ω. Such a limitation allows to associate the ratio between the DToAs to the ratio between the DDoAs among the sensors along the different directions and neglect the propagation velocity.

By posing again $\eta =d_{21}/d_{31}$, the two terms of Equation (11) can be written as:

(12) $$\begin{array}{c} f_{d_{21}d_{21}}=\frac{\partial^{2}f}{\partial \eta^{2}}\frac{1}{{\hat{d}}_{31}^{2}}\\ f_{d_{31}d_{31}}=\frac{1}{{\hat{d}}_{31}^{2}}\left(\frac{\partial^{2}f}{\partial \eta^{2}}\eta^{2}+\frac{\partial f}{\partial \eta }2\eta \right) \end{array}$$

Then, the first equation of (11) becomes:

(13) $$\begin{array}{c} \frac{f^{\prime \prime }\left(1+\eta^{2}\right)+f^{\prime }2\eta }{{\hat{d}}_{31}^{2}}=0 \end{array}$$

where $f^{\prime }=\frac{\partial f}{\partial \eta }$ and $f^{\prime \prime }=\frac{\partial^{2}f}{\partial \eta^{2}}$. We can assume that $d_{31}\neq 0$. Observe that Equation (13) requires just 1D-boundary conditions. Then, by integrating Equation (13), we obtain:

(14) $$\theta =f\left(\frac{d_{21}}{d_{31}}\right)=c_{1}\mathrm{atan}\left(\frac{d_{21}}{d_{31}}\right)+c_{2}$$

where $c_{1}$ and $c_{2}$ are constants to be determined by taking into account the boundary conditions, namely the range of θ. Equation (14) is the extremal function, i.e., the candidate function to minimize the functional (6). This results in the selection of the Mean Square Error (MSE) estimator in the form:

(15) $$\hat{\theta }=f\left(\frac{{\hat{d}}_{21}}{{\hat{d}}_{31}}\right)=c_{1}\mathrm{atan}\left(\frac{{\hat{d}}_{21}}{{\hat{d}}_{31}}\right)+c_{2}$$

Observe that the CoV, when applied to a realistic physics problem, typically provides an extremant function. In our context, the aim is related to the physics of waves and to find their DoA with minimum uncertainty; for this reason, the function (14) is an extremant function for the functional (6). Furthermore, it can be observed that (15) is the optimal estimation function in terms of minimum variance of worst case. Then, our goal is to find the two functions $d_{21}\left(\theta \right)$ and $d_{31}\left(\theta \right)$ which minimize the functional (9). By computing the $\sigma_{\hat{\theta }}^{2}$ and $\sigma_{\hat{\theta }-WorstCase}^{2}$, considering the estimator (15), the followings results are obtained:

(16) $$\begin{array}{c} \sigma_{\hat{\theta }}^{2}=\frac{1}{c_{1}^{2}}{\left.\frac{{\hat{d}}_{21}^{2}\sigma_{{\hat{d}}_{31}}^{2}+{\hat{d}}_{31}^{2}\sigma_{{\hat{d}}_{21}}^{2}}{{\left({\hat{d}}_{21}^{2}+{\hat{d}}_{31}^{2}\right)}^{2}}\right|}_{E\left[d_{21}\right],E\left[d_{31}\right]}\\ \sigma_{\hat{\theta }-WorstCase}^{2}={\left.\frac{1}{c_{1}^{2}}\frac{\sigma_{d-Max}^{2}}{{\left(d_{21}^{2}+d_{31}^{2}\right)}^{2}}\right|}_{E\left[d_{21}\right],E\left[d_{31}\right]} \end{array}$$

It must be noticed that considering two functions $E\left[{\hat{d}}_{21}\left(\theta \right)\right]=d_{21}\left(\theta \right)$, $E\left[{\hat{d}}_{31}\left(\theta \right)\right]=d_{31}\left(\theta \right)$ which are limited in absolute value to a maximum value $d_{max}$ (namely that the DDoAs, $\rho_{21}\left(\theta \right)$ and $\rho_{31}\left(\theta \right)$, are limited in absolute value to a maximum distance *r*), the two functions which minimize the $\sigma_{\hat{\theta }-WorstCase}^{2}$ are a sine and cosine function with the same upper and lower bounds, i.e., r/v.

**Figure 1:**
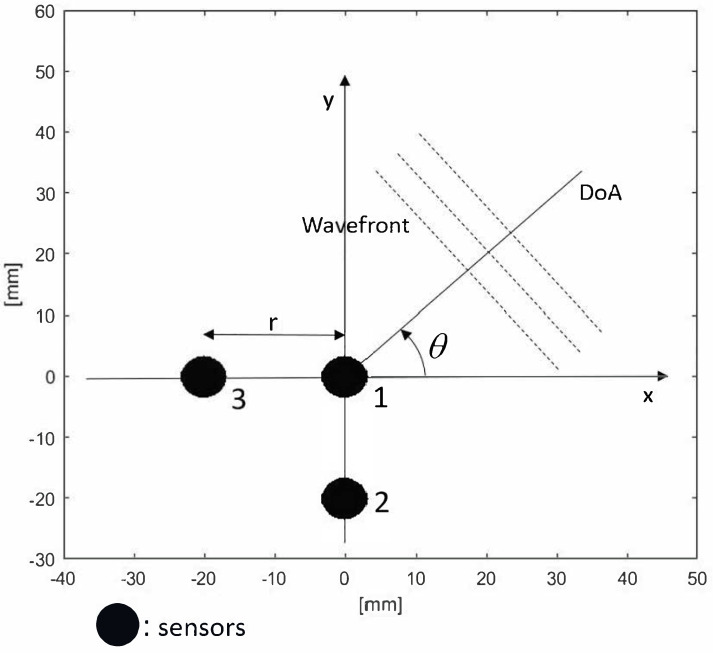
Standard Cluster (SC) of circular sensors.

A Standard Cluster (SC) of three disk sensors is shown in Figure 1, corresponding to the MSE estimator in (15), with the values of the constants $c_{1}=1,\;c_{2}=0$. This cluster configuration was validated by Kundu et al. [14]. In this case, we have that:

(17) $$d_{21}\left(\theta \right)=\frac{r\sin\theta }{v}\;\;\;\;\;\;d_{31}\left(\theta \right)=\frac{r\cos\theta }{v}$$

where *r* is the distance between the sensors. Using (4) and (15), we can calculate the variance (and its value of worst case) of the SC corresponding estimator as:

(18) $$\begin{array}{c} \sigma_{\hat{\theta }}^{2}={\left.\frac{{\hat{d}}_{21}^{2}\sigma_{{\hat{d}}_{31}}^{2}+{\hat{d}}_{31}^{2}\sigma_{{\hat{d}}_{21}}^{2}}{{\left({\hat{d}}_{21}^{2}+{\hat{d}}_{31}^{2}\right)}^{2}}\right|}_{E\left[d_{21}\right],E\left[d_{31}\right]}\\ \;\;\;\;\;\;\;={\left(\frac{v}{r}\right)}^{2}\left(\sin^{2}\left(\theta \right)\sigma_{{\hat{d}}_{31}}^{2}+\cos^{2}\left(\theta \right)\sigma_{{\hat{d}}_{21}}^{2}\right)\\ \sigma_{\hat{\theta }-WorstCase}^{2}={\left(\frac{v}{r}\right)}^{2}\sigma_{d-Max}^{2} \end{array}$$

The SC provides an optimal solution when the full (360°) angular range is monitored. However, in many realistic scenarios, just a DoAs sector has to be monitored, e.g., a quadrant of 90°. In this case, the two constants can be set to $c_{1}=1/2$ and $c_{2}=0$, and the optimal functions $d_{21}\left(\theta \right)$, $d_{31}\left(\theta \right)$ are given by:

(19) $$d_{21}\left(\theta \right)=\frac{r\sin2\theta }{v}\;\;\;\;\;\;d_{31}\left(\theta \right)=\frac{r\cos2\theta }{v}$$

The functions (19) define our Optimal Cluster of Shaped Sensors (SS-OC). Again, by applying (4) to (15), the $\sigma_{\hat{\theta }}^{2}$ and $\sigma_{\hat{\theta }-WorstCase}^{2}$ can be computed:

(20) $$\begin{array}{c} \sigma_{\hat{\theta }}^{2}=\frac{1}{4}{\left.\frac{{\hat{d}}_{21}^{2}\sigma_{{\hat{d}}_{31}}^{2}+{\hat{d}}_{31}^{2}\sigma_{{\hat{d}}_{21}}^{2}}{{\left({\hat{d}}_{21}^{2}+{\hat{d}}_{31}^{2}\right)}^{2}}\right|}_{E\left[d_{21}\right],E\left[d_{31}\right]}\\ =\frac{1}{4}{\left(\frac{v}{r}\right)}^{2}\left(\sin^{2}\left(2\theta \right)\sigma_{{\hat{d}}_{31}}^{2}+\cos^{2}\left(2\theta \right)\sigma_{{\hat{d}}_{21}}^{2}\right)\\ \sigma_{\hat{\theta }-WorstCase}^{2}=\frac{1}{4}{\left(\frac{v}{r}\right)}^{2}\sigma_{d-Max}^{2} \end{array}$$

Observe that the worst-case variance in Equation (20) was reduced four times with respect to the worst-case variance of Equation (18) achievable by a SC. Under a realistic hypothesis, such as signal and noises mutually independent, zero mean stationary Gaussian random processes and equal noise spectra, the time delay variances are equal, when an efficient estimator is used for the DToAs estimation (see [24]). Therefore, $\sigma_{\hat{\theta }-WorstCase}^{2}=\sigma_{\hat{\theta }}^{2}$. This involves that the DoA estimation performance, expressed in terms of DoA estimator variance, which was computed neglecting the time delay covariance terms, is constant with respect to the DoA for the SC case (see (18)). This last property is true also for the SS-OC (see (20)). The reason is due to the boundary conditions, which were implicitly selected by fixing the constant values $c_{1}$ and $c_{2}$, i.e., η(−π/4)=−∞ and η(π/4)=∞. As result, it is possible to reduce the variance of the estimation by acting on the trade-off uncertainty–work range of DoA. Then, the reduction factor of the variance in the worst case is equal to the reduction factor of the work-range (for the SS-OC, it is possible to work within (−π/4,π/4)).

It is noteworthy that the DoA variance is constant also when the time delay covariance terms are not neglected both for the case of wave velocity known and unknown, and the optimal disks cluster is given by an equilateral triangle (see [23,32]).

In the following section, we will exploit a sensor re-shaping procedure able to impose the same frequency response (i.e., the same wave-number tuning effect) among all sensors, when an incoming Guided Wave mode impinges from a given DoA θ, except for phase shifts linearly dependent on DToAs (19). In this way, it is still possible to use GCC procedures to estimate the DToAs between pairs of sensors in order to extract the desired DoA information.

## 3. A Radon Transform-Error Diffusion Re-Shaping Procedure for GWs DoA Estimation

Following the previous works [25,26], a technique based on the math-tool called Radon Transform will be employed to impose the desired time delays and the same wave-number filtering among differently shaped sensors. However, unlike the previous works, a more refined quantization technique, called Error Diffusion, will be exploited. It consists of using dithered piezo-load sensors so that functions continuously modulated in values can be emulated, allowing us to better achieve the desired frequency responses.

In order to proceed, given the frequency response of a disk sensor $V_{P1}\left(\omega ,\theta \right)$, we need to impose that the frequency responses of sensors P2 and P3 ($V_{P2}\left(\omega ,\theta \right)$ and $V_{P3}\left(\omega ,\theta \right)$) are linked to the previous one in the following form:

(21) $$\begin{array}{c} V_{P2}\left(\omega ,\theta \right)=V_{P1}\left(\omega \right)\exp\left(-jk_{0}\left(\omega \right)\rho_{21}\left(\theta \right)\right)\\ V_{P3}\left(\omega ,\theta \right)=V_{P1}\left(\omega \right)\exp\left(-jk_{0}\left(\omega \right)\rho_{31}\left(\theta \right)\right) \end{array}$$

where $\rho_{21}\left(\theta \right)$ and $\rho_{31}\left(\theta \right)$, namely the DDoAs, related to the DToAs via Equation (2), are given by the numerators of Equation (19). With no lack of generality, the term $k_{0}\left(\omega \right)$ is used to indicate the wave-number–frequency relationship of a 0 order Lamb Wave mode. Indeed, according to the derivation of the frequency response model considered in this work (see Section 3.1), the term $k_{0}\left(\omega \right)$ can be replaced by any other wave-number–frequency relationship, k(ω), related to higher Lamb Wave mode orders or also to different GWs which can propagate in thin-wall plates, such as Lamb–Rayleigh waves or Shear Horizontal waves [1].

It is important to observe that the phase-shifts in (21) are in $k_{0}\left(\omega \right)$, instead of ω. Therefore, in Equation (21), we must consider that the P2 and P3 responses may differ from the P1 response not only because of the time-shifts but also for the detrimental effect of wave dispersion. However, if the acquired signal is narrow-band, $k_{0}\left(\omega \right)$ can be approximated via a first-order Taylor expansion around the central frequency $\omega_{c}$:

(22) $$\begin{array}{c} k_{0}\left(\omega \right)≃k_{0}\left(\omega_{c}\right)+\left(\omega -\omega_{c}\right){k_{0}}^{\prime }\left(\omega_{c}\right)\\ \;\;\;\;\;\;\;\;\;\;\;\;\;\;\;=v_{p}\left(\omega_{c}\right)\omega_{c}-\omega_{c}{k_{0}}^{\prime }\left(\omega_{c}\right)+\omega {k_{0}}^{\prime }\left(\omega_{c}\right)\\ \;\;\;\;\;\;\;\;\;\;\;\;\;\;\;=v_{p}\left(\omega_{c}\right)\omega_{c}-\omega_{c}{k_{0}}^{\prime }\left(\omega_{c}\right)+\frac{\omega }{v_{g}\left(\omega_{c}\right)} \end{array}$$

Therefore, for a narrow-band signal, the expansion (22) well approximates the curve $k_{0}\left(\omega \right)$ around the central (angular) frequency $\omega_{c}$, and a phase shift in the frequency ω, equal to $d_{21}\left(\theta \right)=\rho_{21}\left(\theta \right)/v_{g}\left(\omega_{c}\right)$, is achieved, and the wave velocity can be considered approximately constant. It is noteworthy that the closer the sensors are, the smaller the phase shift differences of the spectral components are among all sensors.

The assumption of a narrow-band signal is realistic for impact signals. Conversely, Acoustic Emissions (AEs) due to defects growth can be wide-band. Although efficient dispersion compensation procedures can be applied [33], they require the knowledge of the dispersion curves. Alternatively, the acquired signal can be decomposed, via filter banks, in multiple narrow-band signals. For these signals, the propagation wave velocity $v_{g}$ can be considered nearly constant, and cross-correlation procedures can be computed multiple times to estimate the time delays in each band. Therefore, for wide-band signals, the DoA can be estimated many times to have a more robust estimation to the noise and no-dispersion affected (see [34]).

In conclusion, the dispersion effect can be assumed to be negligible both for narrow-band and wide-band signals and the derived time delays (19) are optimal for any structure without assuming an *a priori* knowledge of the material properties (dispersion curve).

In order to impose the relationships (21), the following four key elements will be exploited:

(a) The model of the frequency response of a sensor in the presence of a Lamb Wave mode.
(b) The Radon Transform and its inverse.
(c) The Projection Slice Theorem.
(d) The error diffusion quantization technique.

In the following subsections, they will be briefly summarized and finally exploited to achieve our aim, which is expressed by Equation (21).

### 3.1. Sensor Frequency Response Model in Presence of a Lamb Wave Mode

For the piezo-load frequency response, the model proposed by Senesi and Ruzzene in [35] is adopted:

(23) $$V_{P}\left(\omega \right)=jU\left(\omega \right)k_{0}\left(\omega \right)H_{P}\left(\theta \right)D_{P}\left(\omega ,\theta \right)$$

where U(ω) denotes the amplitude and the polarization of the wave component relevant to the piezo properties of the patch at the considered frequency, $k_{0}\left(\omega \right)$ is the wave vector of the propagating 0 order Lamb Waves mode, $H_{P}\left(\theta \right)$ is a quantity related to the material properties, and finally, $D_{P}\left(\omega ,\theta \right)$ is the sensor Directivity function, which is the only function which is dependent on the shape of the sensor. It can be computed by the following integral:

(24) $$D_{P}\left(\omega ,\theta \right)=\int_{\Omega_{P}}e^{-jk_{0}\left(\omega \right)\left(x\cos\theta +y\sin\theta \right)}\phi_{P}\left(x,y\right)dxdy$$

where $\phi_{P}\left(x,y\right)$ is referred to as a shape function and describes the shape of the sensor as a step function of form:

(25) $$\phi_{P}\left(x,y\right)=\left\{\begin{array}{c} 1\;\;\;\;\;\left(x,y\right)\in \Omega_{P}\\ 0\;\;\;\;\left(x,y\right)\notin \Omega_{P} \end{array}\right.$$

where $\Omega_{P}$ is the area of the piezoelectric path. Without lack of generality, here, we consider the case of sensors with constant piezoelectric properties.

Therefore, according to the model (23), assuming the same AS signal spectrum U(ω) the sensors frequency responses are determined only by their Directivity functions. From Equation (24), the Directivity function can be computed as the *bi-dimensional Fourier Transform* (2D-FT) *at the angle θ* of the shape function $\phi_{P}\left(x,y\right)$. Furthermore, the output variable is evaluated in $k_{0}\left(\omega \right)$: the wave vector of a 0-order Lamb Wave mode impinging on the sensors (e.g., $A_{0}$ or $S_{0}$).

### 3.2. The Radon Transform and Its Inverse

The Radon Transform (RT) [36], in its so-called “normal definition”, is used in many scientific fields, e.g., tomography, astronomy and microscopy [37], where the transformed function, in our case $\phi_{P}\left(x,y\right)$, has no preferred orientation defined. Let us consider a line on its normal form:

(26) ρ=xcosθ+ysinθ

where θ is the angle between the normal segment to the line, going through the reference system origin, whereas ρ is the length of the normal segment. By using the line equation in the form (26), the RT of a considered function $\phi_{P}\left(x,y\right)$ is given by:

(27) $$RT_{\theta }\left(\rho \right)\left[g\left(x,y\right)\right]=\int \int_{}\phi_{P}\left(x,y\right)\delta \left(\rho -x\cos\theta -y\sin\theta \right)dxdy$$

**Figure 2:**
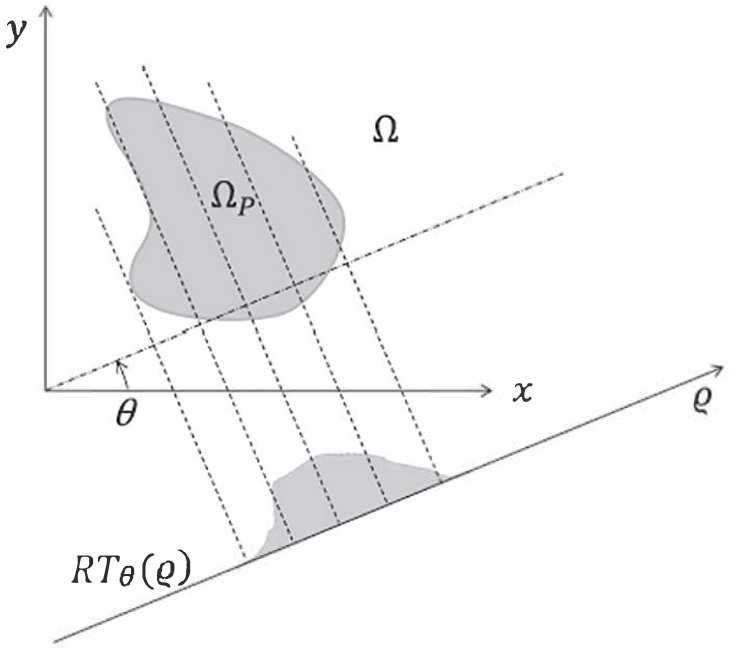
Representation of the Radon Transform of the function $\phi_{P}\left(x,y\right)$ over the domain $\Omega_{P}$.

From the definition (27), it can be observed that the RT consists of multiple integrals along lines shifted by ρ and normal to the direction θ. A graphic illustration is given in Figure 2. It can be observed that the line integrals are computed in the normal direction with respect to to the angle θ. This aspect will be useful for the following section.

As final note, observe that the RT can be inverted. The Inverse RT will be referred to as IRT.

### 3.3. The Projection Slice Theorem

**Figure 3:**
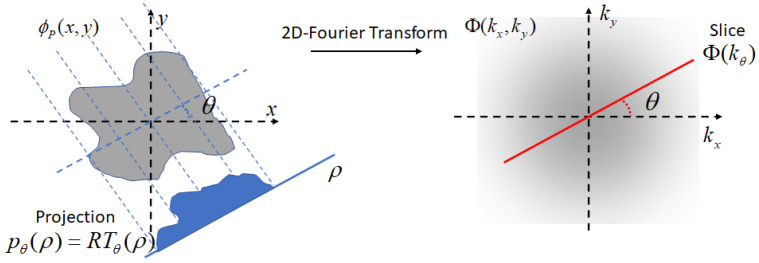
A graphical illustration of the Projection Slice Theorem. $\Phi (k_{x},k_{y})$ is the 2D-FT of ϕ(x,y). The projection given by the RT, $p_{\theta }\left(\rho \right)$, is given by multiple line integrals along lines normal to θ direction. The slice $\Phi (k_{\theta })$ on the 2D-Fourier plain is along the θ direction. The Projection Slice Theorem states that the slice $\Phi (k_{\theta })$ is the 1D-FT of the projection $p_{\theta }\left(\rho \right)$.

The Projection Slice Theorem [38] states that the bidimensional FT at the angle θ of a given function is equal to the monodimensional FT of the Radon Transform of that function. A graphical illustration of the projection slice theorem is given in Figure 3.

Note that as an immediate consequence, the Directivity function, and, in turn, the frequency response, can simply be calculated as the monodimensional FT of the RT at angle θ of the shape function and by evaluating into $k_{0}\left(\omega \right)$.

It is interesting to observe that only the 1D-function of the RT at angle θ (i.e., the “projection”) determines the wave-number filtering effect, i.e., what wavelengths are integrated by producing charge via a piezoelectric effect. In other words, the RT at angle θ acts as an equivalent 1D piezo-load distribution able to provide the effect of a 2D piezo-load distribution on the wave-number/frequency response. It is worth noting that all the information provided by the RT function at θ is computed via line integrals which are normal to the DoA.

### 3.4. The Error Diffusion Quantization Technique

The DToAs relations between the two pairs of sensors (19), or the DDoAs, $\rho_{21}\left(\theta \right),\rho_{31}\left(\theta \right)$, given by the numerators of Equation (19), can be realized through shaped sensors characterized by shape functions which are ideally continuously modulated in values, as it will be shown in the next section. If a simple binary quantization procedure is applied on the ideal shape functions, step sensor–shape functions on compact space areas are obtained. Unfortunately, this strategy is incapable of achieveing the desired non-linear trends in terms of DDoAs due to quantization non-idealities.

**Figure 4:**
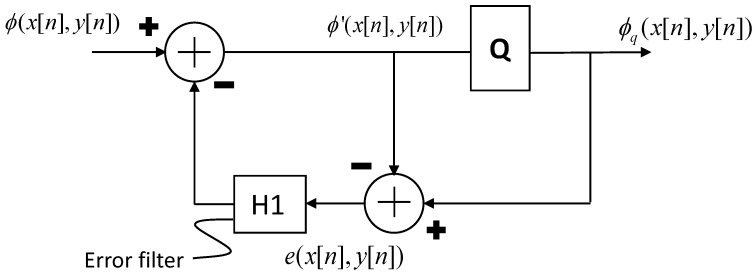
An illustrative scheme of the *Halftoning-Error Diffusion* quantization technique. The ideal piezo-shape function normalized on its maximum value is discretized and considered as input. Each discrete current value is set to 1 or 0 (binary quantized). The quantization error is algebraically summed to the current value and is brought in feedback, filtered via the Error filter H1, and subtracted to the new current value.

In order to address this problem, techniques developed in image processing to quantize gray-scale images with a bit per pixel can be fruitfully adopted. In particular, the so-called *dithering* or *half-toning* techniques can be used (see the works [39,40]). Among these, the Error Diffusion technique is able to work on image sub-areas. Therefore, what the algorithm does at one location influences what happens at other locations. Other simpler techniques do not have these complications but have worse performance in terms of “similarity” parameters with respect to the starting image. In more detail, in the error diffusion approach, the image is scanned, the pixel is quantized and the quantization error is subtracted from the adjacent pixels on the basis of the coefficients of a predetermined filter (error filter). A graphical illustration of the previous procedure is given in Figure 4. In the following, the optimal error filter H1 (see [41]) will be used. It is given by

(28) $$H1=\frac{1}{16}\left[\begin{array}{c} -\;\;\;\;\#\;\;\;\;\;7\\ 3\;\;\;\;\;5\;\;\;\;\;\;1 \end{array}\right]$$

where “−” denotes a point in the current row which has already been processed (hence diffusing error to it would be pointless), and “#” denotes the pixel currently being processed.

### 3.5. Piezo-Loads Synthesis Procedure

On the basis of the Projection Slice Theorem, Equation (21) in the frequency domain, with the desired DDoAs $\rho_{21}\left(\theta \right)$ and $\rho_{31}\left(\theta \right)$, is obtained by imposing the following two relations between the RT of a reference disk sensor P1 and the RT of the shaped sensors P2 and P3:

(29) $$\begin{array}{c} \begin{array}{c} RT_{\theta }\left(\rho \right)\left[\varphi_{2}\right]=RT_{\theta }\left(\rho -\rho_{21}\left(\theta \right)\right)\left[\varphi_{1}\right] \end{array}\\ \begin{array}{c} RT_{\theta }\left(\rho \right)\left[\varphi_{3}\right]=RT_{\theta }\left(\rho -\rho_{31}\left(\theta \right)\right)\left[\varphi_{1}\right] \end{array} \end{array}$$

In this way, the desired time delays $d_{21}\left(\theta \right)=\rho_{21}\left(\theta \right)/v$, $d_{31}\left(\theta \right)=\rho_{31}\left(\theta \right)/v$ are achieved thanks to the FT and RT relation and the FT translation/time shifting property.

**Figure 5:**
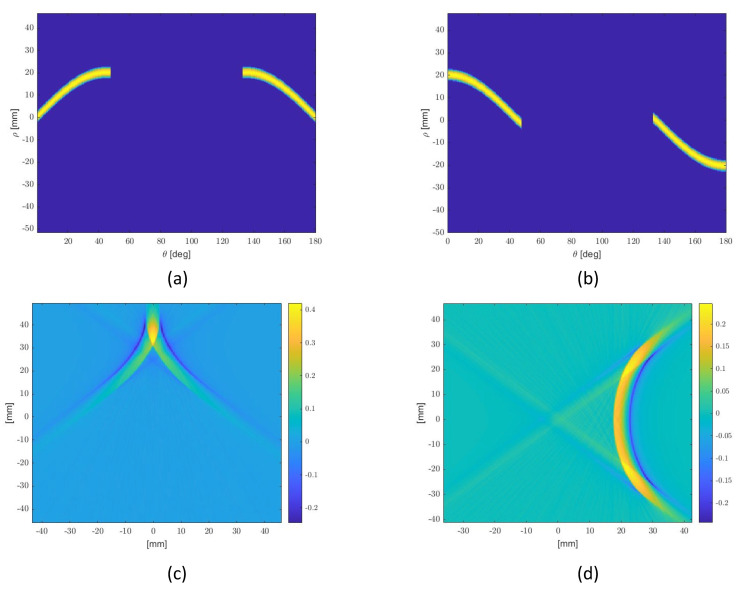
A graphical illustration of the re-shaping design procedure. P2 and P3 are related to the first and second column respectively: (**a**,**b**) Imposed RTs, equal to disk one in [−45°, 45°], unless there are shifts in θ (according to the numerators of Equation (19)); (**c**,**d**) IRTs: ideal shape functions.

In conclusion, the synthesis procedure of the shape functions for P2 and P3, which realize the desired DToAs relation (19), can be summarized as follows: consider an isotropic circular reference sensor P1; then, calculate its RT (constant over θ) and impose the RT of sensors P2 and P3 by using (29) with the desired shifts: $\rho_{21}\left(\theta \right)=r\sin2\theta$ and $\rho_{31}\left(\theta \right)=r\cos2\theta$ (see Figure 5a,b). By inverting the imposed RTs, two shape functions are obtained, which are continuously modulated (Figure 5c,d).

In order to emulate the ideal piezo-loads, the Error Diffusion quantization technique can be applied on the P2 and P3 sensors shape functions.

Although the experimental validation is beyond the scope of this paper, it is worth mentioning that the proposed cluster of shaped sensors can be realized by relying on different piezoelectric materials and manufacturing techniques, e.g., metallized PVDF (polyvinylidene fluoride) sheets can be used by shaping the electrodes on the upper surface with a laser cut as in [42]. Alternatively, the shaping strategy can be based on printing metallic electrodes on PVDF films to obtain the desired shape sensors as proposed in [43] or by using litographic procedures as in [44]. Screen printing was used also to realize shaped PZT transducers [45].

In the practical adoption of these devices, a fundamental step is the definition of a well-controlled bonding procedure, because it may heavily affect the sensor response [46].

Among the last techniques, the PZT/PVDF screen printing was considered for its relative good resolution, near 200/250 μm, and, at the same time, to limit the manufacturing cost.

Therefore, the design procedure was computed to achieve “dots-shape” functions with a resolution of 250 μm to satisfy the limitations that are associated with the patch manufacturing.

The half-toning technique was applied according to the following steps:

1. The sensor shape functions are divided in two parts: positive and negative values.
2. In order to limit the physical sensors size, for each part, the absolute values lower than 25% of the maximum absolute value are discarded.
3. The Error Diffusion (Section 3.4) technique is applied on each part. As a result, each sensor is composed of two parts. However, just a single differential acquisition channel is required; namely, no additional hardware complexity is required.

**Figure 6:**
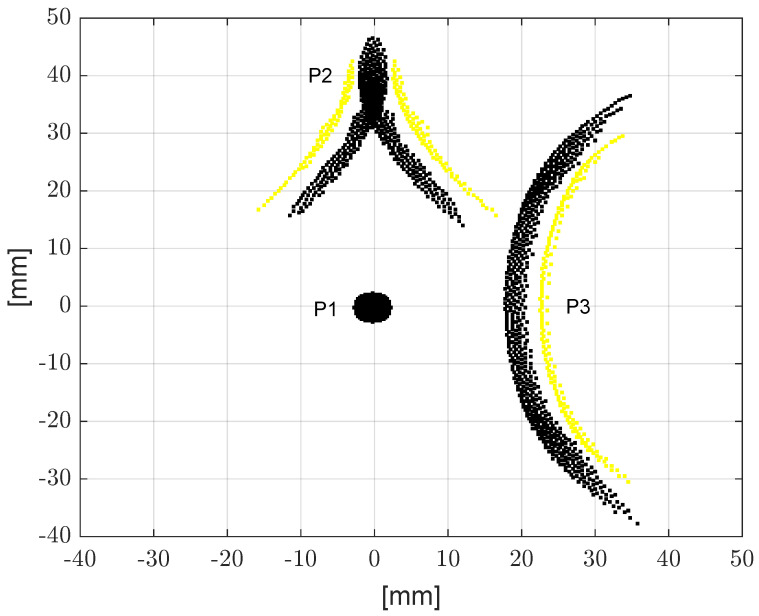
The piezo-sensors P1, P2 and P3 of the Shaped Sensors Optimal Cluster, after applying the Error Diffusion quantization technique (in black color, the positive sensors shape function parts, in yellow color, the negative sensors shape function parts).

The resulting SS-OC is illustrated in Figure 6. It is worth noting that the increased performance is not due to higher distances between sensors. Indeed, the maximum distance between sensors, which defines the maximum time-window duration and how much the dispersive effect influences the acquired signals, can be defined in the RT domain via the maximum DDoA. Because in the RT domain the same maximum distance of disks sensors is imposed, the comparison of two clusters is provided with the same computational cost (i.e., the same maximum duration of time-windows to be stored and processed) and with the same detrimental dispersion effect.

**Figure 7:**
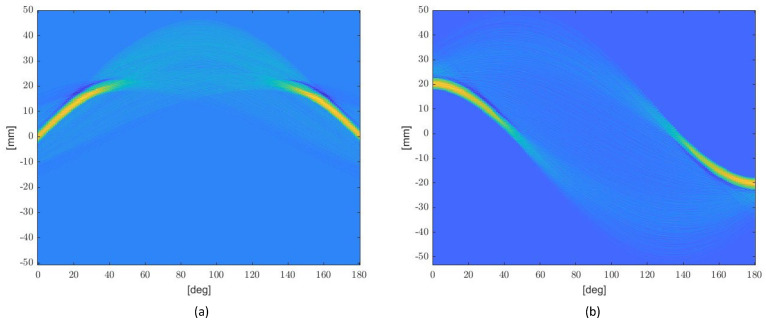
The Radon Transform of the sensors after applying the error diffusion quantization technique: (**a**) Actual RT of P2; (**b**) Actual RT of P3.

Figure 7a,b shows the actual RTs (i.e., the RTs computed by the post-quantization shape functions). It is worth noting that they slightly differ from the imposed ones (Figure 5c,d) due to the quantization procedure.

## 4. Doa Estimators

Given the “true” relation between the DoA and the time-delays ratio for the considered clusters of Figure 1 and Figure 6, the Mean Square Error (MSE) estimators of θ are given by the estimation functions:

(30) $${\hat{\theta }}_{SC}=f\left(\frac{{\hat{d}}_{21}}{{\hat{d}}_{31}}\right)=\mathrm{atan}\left(\frac{{\hat{d}}_{21}}{{\hat{d}}_{31}}\right)\;\;\;\;\;;\;\;\;\;\;{\hat{\theta }}_{SS-OC}=f\left(\frac{{\hat{d}}_{21}}{{\hat{d}}_{31}}\right)=\frac{1}{2}\mathrm{atan}\left(\frac{{\hat{d}}_{21}}{{\hat{d}}_{31}}\right)$$

Considering a small perturbation analysis, when an efficient time-delays estimator is used, their variances attain the CRBs of the DoA, considering the wave velocity unknown. It is important to note that the variances $\sigma_{\hat{\theta }}^{2}$, according to a perturbative approach as in (18) and (20), depend on the time delays–DoA relations, i.e., by the array design, and on the time-delays variances, which, in turn, depend by signal and noise spectra and the employed DToAs estimator.

In the previous work [26], three different estimation modalities were considered for the two DToAs ($d_{21},d_{31}$) estimation. In this work, the last ones will be re-considered together with a new fourth modality, as follows:

1. Locating the peaks of two cross-correlation (CC) products;
2. By using the Gauss–Markov (GM) estimator consisting in three cross-correlation procedures;
3. Locating the peaks of two Generalized Cross-Correlation (GCC) products (filtering first the signals with an optimal filter);
4. By combining the Gauss–Markov estimator with three GCC procedures;

Although a simple cross-correlation procedure implements a Maximum Likelihood (ML) estimator approximation (see [47]) whose variance tends to the CRLB of the time delay between two sensors (for measurements affected by Additive Uncorrelated Gaussian Noise (AUGN)), in the general case of the *M* sensors array, *M* CC products do not attain the time delays CR Matrix-Bound. This happens mainly because the last method does not exploit the additional information which can be provided by the CC products between all sensors pairs. Furthermore, an ML estimator between just two sensors requires firstly filtering the signals (see [30]). For these reasons, the first modality based simply on two cross-correlations, discarding the additional information of the $d_{32}$ time-shift estimation and not filtering the acquired signals, results in a sub-optimal method.

Despite this, observe that the employed design procedure discards the covariance terms. Indeed, the mean of $\sigma_{\hat{\theta }}^{2}$ on the [−π/4,π/4] angle sector, i.e., the functional (6) or (9), is minimized by considering firstly $\sigma_{{\hat{d}}_{32}}^{2},\sigma_{{\hat{d}}_{23}}^{2}=0$, in Equation (4). This implies that $d_{21}\left(\theta \right)$ and $d_{31}\left(\theta \right)$, functions of the SS-OC, are optimal when each time delay is estimated via a single CC product (i.e., independently by the other ones) or the sensor noises are mutually correlated (so that $\sigma_{{\hat{d}}_{32}}^{2},\sigma_{{\hat{d}}_{23}}^{2}=0$). In other words, in the last two cases, the defined sensor shapes and positioning of the SS-OC allow providing the maximum information on θ (defined as the inverse of the integrand function in Equation (9)) when the wave velocity is unknown.

As result, the adopted design procedure allowed achieving the shaped sensor array for the DoA estimation with a minimum variance, with respect to any other sensor configurations, when the sensors are affected by mutually correlated noises, such as jamming noise, or when only two time delays are estimated, relying just on two cross-correlation products. This property can be advantageously exploited to adopt an estimator with a reduced computational cost.

Differently, the second modality consists of measuring the DToAs for all possible sensors pairs by cross-correlations and then calculating the Gauss–Markov (GM) (weighted) estimate of the DToAs with respect to the first sensor (see [30]). Assuming signal and noises to be mutually independent, zero mean Stationary Gaussian Random Processes (SGRPs), and noises having the same spectrum for each sensor (which is fully satisfied when the signals are acquired by the same device and the main noise contribution is due to the electronic noise), the Gauss–Markov estimator coefficients, for the case of three sensors array, are given by:

(31) $$\left[\begin{array}{c} d_{2GM}\\ d_{3GM} \end{array}\right]=\frac{2}{3}\left[\begin{array}{c} 1\;\;\;\;\;\;\;\;\;\;\;1/2\;\;\;\;\;\;\;\;-1/2\\ 1/2\;\;\;\;\;\;\;1\;\;\;\;\;\;\;\;\;\;\;\;\;\;\;\;1/2 \end{array}\right]\left[\begin{array}{c} d_{12CC}\\ d_{13CC}\\ d_{23CC} \end{array}\right]$$

where $d_{ijCC}$ represents the DToAs between sensor *i* and *j* estimated by using the CC procedures, whereas $d_{iGM}$ represents the time delays with respect to the reference sensor estimated with the GM estimator.

The weights of the Gauss–Markov estimator have a more complex form (expressed by a ratio of time-delays variances) when the noise spectra for each sensor, $N_{i}$, are not all equal or when the signals are pre-filtered via different filters. On the basis of the previous considerations, just a slight improvement in terms of performance is expected. In fact, the SS-OC, discarding the time-delays covariance terms, maximizes the DoA information provided by computing only two CC products at the expense of that one achievable via three CCs. Observe that if three CCs are computed and the measurements are affected by mutually independent noises, the employed design criterion is sub-optimal since it is based on a sub-optimal time-delays covariance matrix $Q_{2CC}$ (i.e., ${\left(Q_{3(G)CC}\right)}_{ii}<{\left(Q_{2(G)CC}\right)}_{ii}$, with i=1,2).

The third modality relies on two generalized cross-correlation procedures. For the considered case that the ratio $S/N_{i}$ is the same at each sensor (i.e., the noise spectra are equal), and under the previous hypothesis of signal and noises being mutually independent, zero mean SGRP, the optimal filter [30] to be applied to the acquired signals is given by:

(32) $${\left|F_{OPT(\omega )}\right|}^{2}=\frac{S\left(\omega \right)/N^{2}\left(\omega \right)}{1+2(S(\omega )/N(\omega ))}$$

where S(ω) and N(ω) are the power spectra of, respectively, no noisy signal and noise. In practice, the optimal filter requires knowledge or estimation of the signal and noise spectra. A simple estimation method consists of measuring the noise spectrum and computing S(ω) by subtracting the noise spectrum from the noisy signal spectrum. However, due to random variations of noise, spectral subtraction can result in negative estimates of the short-time magnitude or power spectrum. Different methods for reducing and removing the distortions due to the rectification process are proposed in [48].

In this paper, for testing the DoA estimation performance with the two clusters of Figure 1 and Figure 6, we assumed equal White Gaussian (zero-mean) Noise (WGN) spectra and a flat signal spectrum within a band $B_{s}$ to emulate the narrow-band impulsive signals due to an impact. In this case, the Optimum Filter (32) is equal to an arbitrary constant within the signal band $B_{s}$ (the system performance is unaffected by filter gain constant) and 0 elsewhere:

(33) $${\left|F_{OPT_{Flat}}\left(\omega \right)\right|}^{2}=\left\{\begin{array}{c} 1\;\;\;\omega \in B_{s}\\ 0\;\;\mathrm{elsewhere} \end{array}\right.$$

The band $B_{s}$, is estimated by using the spectral subtraction technique just on the reference sensor acquired signal, assuming estimating the white noise level with a standard deviation σ equal to half of the mean value. The distortions induced by the rectification of negative values of the estimated power spectrum *S* are neglected. This assumption is justified when the Signal-to-Noise Ratio (SNR) values are sufficiently high, while the non-linear distortions are not negligible when the Signal-to-Noise Ratio decreases. However, observe that for the aim of estimating just the useful signal band $B_{s}$, instead of denoising the signal within the band, the negative values rectification to null values turns out to be irrelevant even for medium–low SNR values.

For equal noise spectra, the Covariance Matrix $Q_{2GCC}$, achieved by using a time-delays estimator based of two GCC products, is given by:

(34) $$Q_{2GCC}=\sigma_{d_{i1}}^{2}\left[\begin{array}{c} 1\;\;\;\;\;\;0\\ 0\;\;\;\;\;1 \end{array}\right]$$

with (see [30]):

(35) $$\sigma_{d_{i1}}^{2}=\frac{2\pi }{T_{s}\int_{0}^{B_{s}}2\omega^{2}\frac{S^{2}/N^{2}}{1+2(S/N)}d\omega }$$

where *S* and *N* are, again, the power spectra of signal and noise, $B_{s}$ is the processed signal band, and $T_{s}$ is the signal time window length. According to the discussion related to the estimator based on two CC products, good DoA estimation performance is expected by using just two GCC procedures.

Finally, the last estimator, i.e., the GM one based on Generalized CC (GCC) procedures, is an ML efficient estimator; namely, its covariance matrix attains asymptotically the DToAs CRMB [30]. For the considered case of equal noise spectra, the last one is given by:

(36) $$Q_{3GCC}=\sigma_{d_{i1}}^{2}\left[\begin{array}{c} 1\;\;\;\;\;\;1/2\\ 1/2\;\;\;\;\;1 \end{array}\right]$$

with:

(37) $$\sigma_{d_{i1}}^{2}=\frac{2}{3}\frac{2\pi }{T_{s}\int_{0}^{B_{s}}2\omega^{2}\frac{S^{2}/N^{2}}{1+3(S/N)}d\omega }$$

where *S*, *N*, $B_{s}$ and $T_{s}$ have the same meaning specified for the case of the two GCC-based estimator. The GM-3GCC estimator is given by the set of Equation (31), where in place of CC products, GCC ones are used.

For a generic number of sensors *M*, the optimal filter to be applied to the acquired signals is given by:

(38) $${\left|F_{OPT}\left(\omega \right)\right|}^{2}=\frac{S\left(\omega \right)/N^{2}\left(\omega \right)}{1+M(S(\omega )/N(\omega ))}$$

Considering the signal and noise modeling previously said for the numerical testing, the optimal filter is simply led back to (33).

As a final note, observe that the last ML estimator addresses only the noise issue. In a realistic scenario, another well-known problem may rise: edge reflections. They could cause the “correct” DoA estimation failure. A Phase Transform applied to the GCCs can be adopted to address the reverberation [49,50]. However, its performance can only be considered optimal for a medium–high SNR (as shown in [51]). In a more recent work, ref. [52], a time-delay estimator based on a low-rank approximation of the sub-bands GCC-Matrix has proposed to address efficiently both the noise and reverberation. However, it has been developed for non-dispersive contexts. Improved performance for a lower SNR in the presence of boundaries reflections can be achieved by using GCC procedures with the Hilbert Transform, as proposed in [53].

## 5. Numerical Validation: DoA Estimation Performance

In order to validate the design procedure of the shaped sensors and the improved performance expected in DoA estimation, a numerical analysis was performed.

In particular, impact waves propagating in an aluminum plate 1 mm thick (Young’s modulus 70 GPa, Poisson’s coefficient 0.3 and material density 2700 Kg/m${}^{3}$) were simulated with the Green’s functions formalism adopted in [54]. The impulse response of a band-pass Butterworth filter (10-th order) with different bandwidths and center frequencies was used in order to simulate the impact signal.

Simulations were performed for multiple impact locations obtained by varying the true DoA with $5^{\circ }$ steps ($\theta =-45^{\circ },-40^{\circ },-35^{\circ },\ldots ,45^{\circ }$), the distance from the reference sensor P1 being 0.8 m. The results achieved by the clusters of Figure 1 and Figure 6 are given in Table 1, Table 2 and Table 3 for different center frequencies and bandwidths of the impact signal and different Peak Signal-to-Noise Ratios (PSNR). To assess the Standard Deviation (SD) of DoA estimations, 200 simulations were performed on the entire $90^{\circ }$ sector. Furthermore, the Maximum Error (ME) over all simulations was considered. The simulations were run simulating the propagation of the $A_{0}$ Lamb mode and considering: (i) circular piezo-sensors with a radius equal to 2.5 mm, (ii) maximum DDoA between sensors *d* equal to 2 cm, (iii) sampling frequency (Fs) equal to 2 MHz.

As shown in Table 1, Table 2 and Table 3, the SD and ME values obtained with the shaped sensors and the time-delays estimators based on two CC procedures or given by the GM-CC are, as expected, smaller with respect to the SC, particularly when the PSNR value decreases. This behavior can be interpreted in the following way. Due to the non-ideal calibration curve (i.e., which differs by ideal 1/2arctan(·) obtained via Equation (19)) achieved by the quantized-shaped sensors, there is an error (*bias*), which changes depending on the considered band, in the estimation function. However, the last disadvantage is overcompensated when the noise increases thanks to the achieved curve, which is more robust with respect to the uncertainties on the estimated time delays (i.e., thanks to the reduced variance (20)). When the time-delays estimators based on two GCCs or on the GM-GCC procedure are employed, the advantage of the shaped sensors is provided on the SD values and more clearly on the ME, particularly for low PSNR values. This trend can be explained according to the following reason. When the noise level arises, its estimation “error” arises together with the signal band $B_{S}$ estimation “error”. When the band is overestimated, more noise, summed to the useful signal, is processed. In all these cases, for low PSNR values, the previous behavior applies. As a result, the DoA estimation maximum error achieved via the SS-OC is smaller than the SC one.

It is very interesting to observe that the DoA estimation performance provided by using just two GCC procedures is quite similar to the one provided by the GM estimator which relies on three GCC products. This behavior allows us to consider the former estimator to limit the computational cost, by excluding a third GCC product, to the benefit of the embedded electronic devices power consumption. It derives from the design procedure which neglected the time-delays covariance terms, and it is in accordance with the considerations previously explained in Section 4.

Due to the wave dispersion of the $A_{0}$ mode, the higher the considered center frequency, the higher the wave (group) velocity *v*. Therefore, according to the variances (18) and (20), which increase as $v^{2}$ increases, worse performances are achieved by both clusters when the center frequency increases. This behavior appears in the SS-OC when the noise increases for the above-mentioned reason.

Conversely, when the signal bandwidth increases, a more impulsive signal is detected and the time-delay uncertainties are smaller. For the considered case of a quasi-flat signal spectrum in a given band $B_{S}$, the DToA variance terms $\sigma_{{\hat{d}}_{i1}}^{2}$ of the covariance matrix, for an optimal time-delays estimator based on a GCC procedure, decrease as $B_{S}^{2}$ decreases (see (35)). Therefore, the two terms, wave velocity and central frequency, have an opposite influence on the DoA estimation performance (compare Table 1 and Table 2, and Table 3, which are characterized by a 10 and 30 kHz bandwidth, respectively).

## 6. Discussion

The numeric results showed improved performance in impacts/defects GWs DoA estimation when a Shaped Sensors Optimal Cluster is employed instead of a Standard Cluster of disks sensors. The theoretical variance reduction factor equal to 4, i.e., 2 in terms of DoA “error” in numerical testing, appears to lower PSNR values when the bias term of the shaped sensors estimator (the second of Equation (30)), induced by the quantization procedure non-idealities, is negligible with respect to the estimator variance $\sigma_{\hat{\theta }}^{2}$ (20). It is worth noting that the increased performance is not due to a higher maximum DDoA between sensors pairs, but it is achieved thanks to the implemented optimal time delays–DoA relations. This means that the DoA performance achieved by the SC and the SS-OC is provided with the same computational cost (i.e., the same maximum duration of signals to be stored and processed via GCC products). Furthermore, neglecting the quantization non-idealities, the clusters comparison takes into account the detrimental effect of dispersion which influences the acquired signals. Indeed, the dispersion effect for each angle θ of the monitored angles sector is defined only by the spatial shifts $\rho_{ij}\left(\theta \right)$ in the Radon Transform domain, which have the equal maximum value in both clusters.

It is interesting to compare the cluster optimization of this work with the one in [24], where just disk sensors were considered. In [24], the optimal disks positioning, for an unknown wave velocity, is defined with the constraint to lie in a circle domain of a fixed radius. In order to compare the numeric results in DoA estimation obtained via the *Disk Sensors Optimal Cluster* (DS-OC), defined in [24], considering a circle radius equal to r= 2 cm, it is important to highlight that in that case, the maximum DDoAs between two disks is about 1.7r and 1.4r, whose average value is 1.55r. If such a maximum DDoA was used in the SS-OC design, it has produced SD values reduced by a factor of 1.55 for low PSNR values. Although, due to the optimization procedure differences, mainly related to considering or not the time-delays covariance terms, an impartial comparison can not be made yet, it is possible to analyze and explain the performance achieved by the two different optimized clusters.

Let us consider, as an example, the same bandwidths, 30–40 kHz and 30–60 kHz, and the DoA estimators based on two CC procedures and on the GM-CC one. Generalized CC procedures-based DoA estimators are not taken into account due the fact that in [24], the noise level is considered to be known. When the CCs-based estimator is considered, fixing a certain maximum DDoA value, i.e., fixing the computational cost, the proposed SS-OC shows clearly its advantages in terms of DoA estimation performance when compared to the DS-OC. The DoA estimation improvement is due to the variance $\sigma_{\hat{\theta }}^{2}$ related to the SS-OC (20), which is minimized considering the time-delays covariance terms equal to 0. This means that a higher information content on the DoA is provided by the two time delays $d_{21},d_{31}$ estimated by two simple CC products.

When the GM-CC estimator is considered, i.e., also the time delay $d_{32}$ is taken into account, the SS-OC shows again better performance with respect to the DS-OC of [24] in the narrow-band 30–40 kHz to low PSNR (when the bias error induced by the shaped sensors is negligible with respect to the estimator variance). Vice versa, a worse DoA estimation performance is shown in the wider band: 30–60 kHz. This behavior can be explained by the shaped sensors RT non-idealities, i.e., to higher wave-number filtering differences in wider bands (namely, sensors “transfer function” differences), among the quantized sensors. Indeed, also when the SS-OC is compared to the SC, by observing the Table 2 and Table 3, for the same “DoA error region” of the SS-OC (e.g., the SD values are within the range 3°–5°), better results are achieved in the case of a narrow-band: 30–40 kHz. Furthermore, observe that the DS-OC was designed differently, considering also the time-delays covariance terms, selected as non-zero and equal to non-diagonal terms of the CRMB, i.e., 1/2 of its diagonal terms (37), under the assumption of noises with same spectrum, which is the considered case for the numeric results to emulate the electronic noise. Therefore, the GM-CC estimator exploits better the information provided by the three estimated time delays.

Disk clusters, on the other hand, defined in a restricted circular area, such as the DS-OC, have the advantage of considering a reduced physical sensor size and optimizing the physical distances among the disks, ensuring better the assumption that the wavefronts impinging on the cluster can be considered locally planar for a considered AS location.

Future developments are aimed to define an optimal cluster of shaped sensors without neglecting the covariance terms to improve even more the DoA estimation performance in the case of electronic noise and when the computational cost can be increased by a third supplementary GCC product.

## 7. Conclusions

In this work, an optimal three-shaped-sensors array design procedure for Guided Waves DoA estimation is proposed. The optimality criterion is based on the minimization of the DoA estimation variance provided by the Propagation of Uncertainty, in an average sense, via the Calculus of Variations. It allows defining the optimal time delays–DoA relations between a reference disk sensor and the other two shaped ones, considering the wave velocity unknown.

Following the re-shaping procedure defined in previous works, based on the Radon Transform tool, the ideal sensor shape functions, able to impose the same wave-number tuning effect among all sensors and the desired time delays–DoA relations, are found. The last procedure allows using cross-correlation products to estimate the time delays between pairs of sensors with the ultimate aim of estimating the DoA.

Unlike previous works, a more refined procedure, derived by the image processing techniques, is applied. It is called Error Diffusion. Able to emulate sensor shape functions continuously modulated in values, it allows reducing the non-idealities of the quantized sensors in the Radon Transform domain.

The theoretical improved performance via the designed Shaped Sensors Optimal Cluster (SS-OC), equal to a 50% reduction of the DoA estimation “error” with respect to a Standard Cluster of disk sensors, is assessed via a numerical validation based on the Green’s functions formalism, particularly to low PSNR values.

A numerical comparison of different DoA estimators, based on two or three (G)CC products, was provided to show how the DoA estimation performance is quite similar when there are either two or three (G)CC procedures and when the measurements are affected by electronic noise. This result was expected for the employed design procedure, which discards the time-delays covariance terms. In other words, the DoA estimation variance was minimized relying on just two no-correlated time delays. This criterion is optimal for measurements affected by environment-correlated noises or when the DoA is estimated through only two (G)CC products for computational cost saving.

A discussion of the numeric results achieved by the SS-OC and by the Disk Sensors Optimal Cluster (DS-OC), proposed in the previous work [24], has highlighted the ability of the SS-OC to provide improved DoA estimation performance with respect to the DS-OC when just two CC products are computed.

To conclude, the SS-OC was designed to overcome the performance in the DoA estimation in a 90° angles sector, with respect to any array configuration of disk or shaped sensors, when the DoA estimation procedure relies on just two (G)CC products, i.e., when low-power consumption electronic devices are used and/or environment-correlated noises are addressed, and when the measurements are characterized by a low PSNR value, i.e., when the estimation bias (induced by quantization effects) is overcome by the estimation variance due to noise.

Future developments are aimed to design shaped sensors considering also the time delays covariance terms for the three time-delays-based DoA estimation and mobile boundary conditions in the Calculus of Variations problem statement.

## Figures and Tables

**Table 1:** Comparison of Standard Deviation (SD) and Maximum Error (ME) values (in degrees) between the arrays of Figure 1 and Figure 6 for noise-affected measurements (AWGN) at different PSNR values. Actuated pulse band: [20–30] kHz.

	Standard Cluster								Shaped Sensors Optimal Cluster							
	**CC**		**GM-CC**		**GCC**		**GM-GCC**		**CC**		**GM-CC**		**GCC**		**GM-GCC**	
PSNR	SD	ME	SD	ME	SD	ME	SD	ME	SD	ME	SD	ME	SD	ME	SD	ME
60 dB	1.1	1.79	1.08	1.79	1.07	1.79	1.06	1.79	2.7	4.53	2.71	4.53	2.69	4.53	2.68	4.53
40 dB	1.54	4.93	1.39	4.04	1.12	2.62	1.09	2.62	2.74	6.24	2.73	6.38	2.68	5	2.68	5
35 dB	2.2	7.41	1.91	6.84	1.18	3.3	1.16	3.3	2.83	7.04	2.79	6.91	2.69	5.48	2.69	5.48
30 dB	3.46	10.3	2.88	10	1.47	10.6	1.41	8.03	3.07	9.64	2.96	9.64	2.71	6.66	2.7	6.31
28 dB	4.21	14.2	3.47	12.7	1.78	13.4	1.64	9.18	3.24	10.4	3.05	10.5	2.75	9.12	2.72	8.96
27 dB	4.64	17.4	3.85	17.4	1.9	13.4	1.77	13.8	3.4	11	3.23	11	2.79	8.87	2.77	9.15
26 dB	5.14	18	4.21	18.4	2.17	14.9	2.01	14.9	3.47	10.2	3.25	11.3	2.88	11.3	2.83	9.99
24 dB	6.3	24.7	5.15	20.9	3.24	24.7	2.81	20.8	3.88	13.2	3.57	13	2.98	15.5	2.91	15.7

**Table 2:** Comparison of Standard Deviation (SD) and Maximum Error (ME) values (in degrees) between the arrays of Figure 1 and Figure 6 for noise-affected measurements (AWGN) at different PSNR values. Actuated pulse band: [30–40] kHz.

	Standard Cluster								Shaped Sensors Optimal Cluster							
	**CC**		**GM-CC**		**GCC**		**GM-GCC**		**CC**		**GM-CC**		**GCC**		**GM-GCC**	
PSNR	SD	ME	SD	ME	SD	ME	SD	ME	SD	ME	SD	ME	SD	ME	SD	ME
60 dB	1.27	2.12	1.21	2.12	1.28	2.12	1.2	2.12	2.7	4.53	2.71	4.53	2.69	4.53	2.68	4.53
40 dB	1.8	6.39	1.6	5.56	1.21	2.65	1.17	2.65	2.74	6.24	2.73	6.38	2.68	5	2.68	5
35 dB	2.68	8.83	2.26	8.83	1.25	3.66	1.23	3.66	2.83	7.04	2.79	6.91	2.69	5.48	2.69	5.48
30 dB	4.24	13.2	3.54	12	1.66	10.9	1.57	10.2	3.07	9.64	2.96	9.64	2.71	6.66	2.7	6.31
28 dB	5.18	17.2	4.31	15.5	1.99	13.7	1.86	12.6	3.24	10.4	3.05	10.5	2.75	9.12	2.72	8.96
27 dB	5.78	21.9	4.72	17	2.49	19.5	2.2	18.1	3.4	11	3.23	11	2.79	8.87	2.77	9.15
26 dB	6.48	22.8	5.28	19.8	2.81	20.6	2.46	15.2	3.47	10.2	3.25	11.3	2.88	11.3	2.83	9.99
24 dB	8	27.8	6.67	27.3	3.65	25	3.21	25	3.88	13.2	3.57	13	2.98	15.5	2.91	15.7

**Table 3:** Comparison of Standard Deviation (SD) and Maximum Error (ME) values (in degrees) between the arrays of Figure 1 and Figure 6 for noise-affected measurements (AWGN) at different PSNR values. Actuated pulse band: [30–60] kHz.

	Standard Cluster								Shaped Sensors Optimal Cluster							
	**CC**		**GM-CC**		**GCC**		**GM-GCC**		**CC**		**GM-CC**		**GCC**		**GM-GCC**	
PSNR	SD	ME	SD	ME	SD	ME	SD	ME	SD	ME	SD	ME	SD	ME	SD	ME
60 dB	1.2	2.13	1.23	2.13	1.23	2.28	1.2	2.28	1.25	2.47	1.22	2.47	1.24	2.71	1.19	2.71
40 dB	1.23	3.21	1.19	3.2	1.21	2.28	1.19	2.28	1.3	3.62	1.27	3.41	1.27	2.86	1.22	2.71
35 dB	1.52	4.86	1.38	4.51	1.19	2.87	1.18	2.87	1.39	4.83	1.34	4.63	1.28	2.86	1.24	2.93
30 dB	2.08	6.8	1.81	5.96	1.24	4.29	1.22	4.29	1.56	5.84	1.47	5.33	1.31	4.01	1.28	4.35
28 dB	2.52	8.84	2.15	8.84	1.4	8.2	1.34	8.2	1.73	5.84	1.59	5.84	1.33	4.99	1.3	5.52
27 dB	2.75	10	2.32	8.37	1.5	7.77	1.42	7.43	1.81	7.27	1.64	7.51	1.35	6.79	1.31	4.53
26 dB	2.98	10.1	2.5	9.79	1.75	9.54	1.58	8.96	1.92	7.27	1.73	6.94	1.42	6.98	1.36	6.81
24 dB	3.63	12.1	3.05	10.8	1.99	11.3	1.79	10.2	2.21	7.98	1.98	8.2	1.6	6.09	1.5	7.07
22 dB	4.44	15.2	3.75	13.5	2.96	17.5	2.57	16.3	2.61	9.53	2.29	9.28	1.9	9.55	1.73	8.63
20 dB	5.54	18.3	4.53	17.2	3.92	20	3.33	20	3.07	10.3	2.64	9.84	2.49	13.2	2.19	13.2

## Data Availability

Not applicable.

## Abbreviations

The following abbreviations are used in this manuscript:

AS	Acoustic Source
AUGN	Additive Uncorrelated Gaussian Noise
AWGN	Additive White Gaussian Noise
CC	Cross-Correlation
CoV	Calculus of Variations
CU	Central Unit
CRB	Cramér–Rao Bound
CRMB	Cramér–Rao Matrix Bound
DDoA	Difference in Distance of Arrival
DoA	Direction of Arrival
DS-OC	Disk Sensors Optimal Cluster
DToA	Difference in Time of Arrival
Fs	Sampling Frequency
FT	Fourier Transform
GCC	Generalized Cross-Correlation
GM	Gauss Markov
GW	Guided Waves
IFT	Inverse Fourier Transform
ME	Maximum Error
ML	Maximum Likelihood
MSE	Mean Square Error
MUSIC	Multiple Signal Classification
PSNR	Peak Signal-to-Noise Ratio
RT	Radon Transform
IRT	Inverse Radon Transform
r.v.	random variable(s)
SC	Standard Cluster
SD	Standard Deviation
SGRP	Stationary Gaussian Random Processes
SHM	Structural Health Monitoring
SNR	Signal-to-Noise Ratio
SS-OC	Shaped Sensors Optimal Cluster
WGN	White Gaussian (zero-mean) Noise
2D-FT	Bi-dimensional Fourier Transform
